# Host Interaction Analysis of PA-N155 and PA-N182 in Chicken Cells Reveals an Essential Role of UBA52 for Replication of H5N1 Avian Influenza Virus

**DOI:** 10.3389/fmicb.2018.00936

**Published:** 2018-05-15

**Authors:** Qiao Wang, Qinghe Li, Tao Liu, Guobin Chang, Zhihao Sun, Zhao Gao, Fei Wang, Huaijun Zhou, Ranran Liu, Maiqing Zheng, Huanxian Cui, Guohong Chen, Hua Li, Xiaoya Yuan, Jie Wen, Daxin Peng, Guiping Zhao

**Affiliations:** ^1^Institute of Animal Sciences, Chinese Academy of Agricultural Sciences, Beijing, China; ^2^State Key Laboratory of Animal Nutrition, Beijing, China; ^3^College of Animal Science and Technology, Yangzhou University, Yangzhou, China; ^4^School of Veterinary Medicine, Yangzhou University, Yangzhou, China; ^5^Department of Animal Sciences, College of Agricultural and Environmental Sciences, University of California, Davis, Davis, CA, United States; ^6^School of Life Sciences and Engineering, Foshan University, Foshan, China

**Keywords:** H5N1 IAV, PA-N155, PA-N182, virus–host interaction, UBA52

## Abstract

PA-N155 and PA-N182 proteins were translated from the 11th and 13th start codon AUG of the RNA polymerase acidic protein (PA) mRNA of H5N1 influenza A virus (IAV), which plays an important role in viral replication. Little is known about the interactions between PA-N155 and PA-N182 and the host proteins. This study investigated the interaction landscape of PA-N155 and PA-N182 of H5N1 IAV in chicken cells while their interacting complexes were captured by immunoprecipitation and analyzed by mass spectrometry. A total of 491 (PA-N155) and 302 (PA-N182) interacting proteins were identified. Gene ontology and pathway enrichment analyses showed that proteins of the two interactomes were enriched in RNA processing, viral processing and protein transport, and proteins related to signaling pathways of proteasome, ribosome, and aminoacy1-tRNA biosynthesis were significantly enriched, suggesting their potential roles in H5N1 IAV infection. Comparative analysis of the interactome of PA, PA-N155, and PA-N182 identified UBA52 as a conserved host factor that interacted with all three viral proteins. UBA52 is a fusion protein consisting of ubiquitin at the N terminus and ribosomal protein L40 at the C terminus. Knockdown of UBA52 significantly decreased the titer of H5N1 IAV in chicken cells and was accompanied with attenuated production of proinflammatory cytokines. Our analyses of the influenza–host protein interactomes identified UBA52 as a PA interaction protein for virus replication.

## Introduction

Influenza A virus (IAV) is responsible for seasonal epidemics and represents a serious threat to public health and the poultry industry (Schrauwen et al., 2014). Avian influenza virus is a single-stranded, negative-sense, segmented RNA virus, with a genome consisting of eight gene segments, including PA, PB1, PB2, NP, HA, NA, M1 and NS1, that encode up to 17 proteins by mRNA splicing or alternative translation initiation (Wise et al., 2009, 2012; Jagger et al., 2012; Muramoto et al., 2013).

The RNA polymerase system of IAV consists of PA, PB1, and PB2 (Vasin et al., 2014). Many studies have explored the biological functions of the PA protein, such as promoter binding, cap binding and endonuclease activity (Yuan et al., 2009). The endonuclease activity enables the virus to cleave host mRNA into fragments for their use in synthesizing viral RNA (Bavagnoli et al., 2015). PA-N155 and PA-N182 were translated from the 11th and 13th start codons (AUGs) of PA mRNA (Muramoto et al., 2013; Aitken et al., 2016). Expression of PA-N155 and PA-N182 is universal among most IAVs including the H1N1, H3N2, H5N1, and H9N2 strains (Gong et al., 2014). The expression levels of the PA-N155 and PA-N182 have little effect on viral polymerase activity, but they have important functions for efficient viral replication. PA-N155 might have important roles in viral pathogenicity in animals (Wise et al., 2009; Muramoto et al., 2013).

Influenza A virus relies on host factors to support its life cycle, including transcription, viral RNA transportation, and packaging (Konig et al., 2010), so the interactions of host and viral proteins have an important impact on virulence (Zhang et al., 2016). Identification and understanding of the host proteins and complexes can identify the host cellular machinery that is involved in the course of infection. Hundreds of human factors have dynamic interactions with H1N1 influenza, as demonstrated through yeast two-hybrid analysis (Shapira et al., 2009). Two hundred and seventy-eight human cellular proteins were identified as interacting with the PA of H5N1 IAV using immunoprecipitation assay combined with liquid chromatography-tandem mass spectrometry (Gao et al., 2017). Protein interactomes between host and several IAVs have been mapped and comparative analyses of these interactomes have uncovered the unique and common host proteins that regulate viral infection (Wang L. et al., 2017). The minichromosome maintenance protein, MCM, interacts with PA and is involved in replication of the viral genome (Kawaguchi and Nagata, 2007). Hsu et al. (2013) identified that PA interacts with HCLS1 associated protein X-1 (HAX1) *in vivo* to impede nuclear transport of PA and hence halt viral replication. IAV–host interactions in chicken cells, however, remain incompletely explored.

In the present study, we investigated the PA-N155 and PA-N182 interactome of H5N1 IAV in chicken cells by affinity purification-MS (AP-MS); 491 (PA-N155) and 302 (PA-N182) interacting proteins were identified in chicken DF1 cells. Of these, 222 were common host proteins shared by PA-N155 and PA-N182, including many crucial genes involved in important biological processes. UBA52 was identified as a conserved host factor that interacted with PA, PA-N155 and PA-N182, and was a promotive factor for virus replication. Our study describes the fundamental landscape of PA-N155 and PA-N182 interacting proteins in host cells and promotes a better understanding of the mechanisms of IAV infection.

## Materials and Methods

### Cells and Virus

Chicken embryonic fibroblast (DF1) cells and Madin–Darby canine kidney (MDCK) cells were cultured in Dulbecco’s modified Eagle’s medium (DMEM) supplemented with 10% fetal bovine serum (Gibco), 100 μg/ml streptomycin and 100 U/ml penicillin at 37°C, under a humidified atmosphere of 5% CO_2_. Highly pathogenic H5N1 strain A/mallard/Huadong/S/2005 (SY) (Tang et al., 2009) was propagated in 10-day-old specific-pathogen-free embryonic chicken eggs. All experiments involving live viruses were performed in biosafe cabinet with HEPA filters in biosafety level 3 laboratory in Yangzhou University, Yangzhou, China.

### Plasmid Construction

*PA-N155* and *PA-N182* genes were amplified by high-fidelity DNA polymerase (Transgen) using cDNA derived from H5N1 virus (A/Chicken/ShanXi/2/2006) as a template. A 3× FLAG tag was inserted into the C terminus of the pcDNA 3.1 vector and the *PA-N155* and *PA-N182* genes were cloned upstream of the FLAG tag using the Seamless Assembly Cloning Kit (CloneSmarter) to make FLAG-tagged C-terminal fusion proteins. All expression vectors were validated by sequencing.

### Cells Transfections

Transfection with plasmids was performed with Lipofectamine 3000 (Life Technologies). Cells were harvested for protein extraction 48 h after transfection. The experiments were repeated three times independently, and cells grown in 3 cm × 10 cm dishes were used for transfection.

### Antibodies

The anti-FLAG, anti-MYC, and anti-β-Actin mouse monoclonal antibodies were obtained from Abmart.

### Protein Co-immunoprecipitation (co-IP) and Western Blotting

Transiently transfected cells were washed twice with PBS and lysed in RIPA buffer (50 mM Tris-HCl, pH 7.4, 150 mM NaCl, 0.25% deoxycholic acid, 1% NP-40, 1 mM EDTA, and 0.5% SDS supplemented with protease inhibitor; Roche). Whole cell lysate was cleared with protein A/G slurry (Millipore) and then incubated with 40 μl anti-FLAG affinity gel (Sigma-Aldrich) for 2 h at 4°C. Immunoprecipitated samples were washed with RIPA buffer four times and twice with 54K buffer (50 mM Tris-HCl, pH 7.4, 150 mM NaCl, 0.25% Triton-100 supplemented with protease inhibitor). FLAG tag associated proteins were eluted with 250 ng/μl FLAG peptide (Sigma) by rocking using a tilted rotator for 2 h at 4°C.

For western blotting, after SDS-PAGE, proteins were transferred to polyvinylidene fluoride membranes. Membranes were blocked and incubated with corresponding antibodies. Proteins were visualized using an Immobilon Western Chemiluminescent HRP Substrate (Millipore).

### MS

Strict experimental controls were used for the MS analysis. Immunoprecipitated samples from empty FLAG-transfected cells (empty FLAG control) and protein complexes that were pulled down by normal IgG (IgG control) were also subjected to MS for identification. All proteins identified in these two sets of controls were excluded as interacting proteins of PA-N155 and PA-N182. Authentic FLAG-precipitated proteins for PA-N155 and PA-N182 were examined in triplicate. Proteins that were enriched by co-IP were separated by SDS-PAGE and the entire lane was cut and sent for tryptic digestion.

Proteins pulled down by anti-FLAG beads were digested with trypsin for 20 h at room temperature. Peptides were extracted twice with 50% aqueous acetonitrile containing 0.1% formic acid, dried in a Speed Vac and then desalted using Sep-Pak C18 cartridges. TMT reagents (Thermo Fisher) were used to label the purified peptides. The TMT labeling reagents in anhydrous acetonitrile were carefully added to the desalted peptides, incubated for 1 h at room temperature, and the reactions were stopped by hydroxylamine. The TMT-labeled peptides were separated by reverse phase (RP) chromatography. The first dimension RP separation by microLC was performed by an Ultimate 3000 System (Thermo Fisher) using a XbridgeC18 RP column (5 μm, 150 Å, 250 mm × 4.6 mm inner diameter; Waters). Mobile phases A (2% acetonitrile, pH adjusted to 10.0 with NH_4_OH) and B (98% acetonitrile, pH to 10.0 as above) were used: 5–8% B, over 5 min; 8–18% B, to 25 min; 18–32% B, to 32 min; 32–95% B, over 2 min; 95% B for 6 min; and 95–5% B over 5 min. The peptides were monitored at 214 nm and 1-min fractions were collected, dried and reconstituted in 20 μl of 0.1% (v/v) formic acid in water for nano-LC-MS/MS analyses. The fractions were further separated on a C18 column (75 μm inner diameter, 150 mm length) with a flow rate of 250 nl/min. Mobile phase A consisted of 0.1% formic acid, and mobile phase B consisted of 0.1% formic acid and 100% acetonitrile. The Orbitrap Q-Exactive mass spectrometer was operated in the data-dependent acquisition mode using Xcalibur 3.0 software. The scan range was from m/z 300 to 1800 with resolution 70,000 at m/z 400. A full-scan followed by 20 data-dependent MS/MS scans were acquired with collision-induced dissociation with normalized collision energy of 35%. The MS/MS spectra from each LC–MS/MS run were searched against the protein database using the Proteome Discovery searching algorithm. Precursor ion mass tolerance was set to be 20 ppm and the fragment ion mass tolerance was 20 mmu. One missed trypsin cleavage event was allowed. The FDR rate of the LC–MS/MS is 1%. Oxidation (Met) was chosen as variable modifications. Carbamidomethyl (Cys) and TMT6plex were chosen as the fixed modifications.

### Protein Interaction Analysis and Functional Enrichment Analysis

All proteins that interacted with PA were entered into The Search Tool for the Retrieval of Interacting Genes/Proteins (STRING; Franceschini et al., 2013) and these interactions were mapped. Only high confidence interactions analyzed by significance analysis of interactome (SAINT; Choi et al., 2012) with confidence score > 0.89 were considered for subsequent analysis. Gene ontology enrichment and pathway enrichment analysis was conducted using the Database for Annotation, Visualization and Integrated Discovery (DAVID; Huang et al., 2009) and the KEGG Orthology Based Annotation System (KOBAS), respectively. Enriched protein domain analysis was done using the Functional Enrichment analysis tool (Funrich). Our data package has been approved, and will be available in the repository once the associated article has been published (doi: 10.5061/dryad.77r8m5g).

### RNA Interference

All the siRNAs used in this study were designed and synthesized by Guangzhou Ruibo (Guangzhou, China). DF1 cells, at a confluence of 90% in 6-well plates, were transfected with 100 nM effective siRNA specific for chicken UBA52 gene (Gene ID: 395958); siUBA52-1, sense 5′-CCAAGAAGAAGGTCAAATA-3′. siUBA52-2, sense 5′-GGAGCCCAATGACACCATC-3′. The negative control siRNA was a scrambled siRNA for UBA52 (siNC, sense 5′-GUGAACGAACUCCUUAAUUTT-3′). All siRNAs were transfected into DF1 cells using Lipofectamine 3000 (Life Technologies).

### Infectious Titer of Influenza Virus (TCID50 Assay)

The TCID50 assay was used to evaluate progeny virus production. After transfection with siRNAs, DF1 cells were infected with the SY virus at a multiplicity of infection of 0.1 PFU. At 1 h post infection (1 hpi), the medium was replaced with DMEM without fetal bovine serum. Conditioned media were collected at 12 and 24 hpi for measuring viral titers. Viral titers were determined by agglutination assay after growth in MDCK cells. The MDCK cells were seeded in 96-well plates and infected after reaching 85% confluence. Cells were washed with phosphate-buffered saline (PBS) twice and infected with a series of dilutions of viruses, and incubated for 72 h, as described above. Agglutination assays were performed in a round-bottomed 96-well plate using 1% chicken red blood cells in PBS.

### Quantitative Real-Time Polymerase Chain Reaction (PCR)

The efficiency of UBA52 expression knockdown was confirmed by quantitative real-time PCR. Total RNA was isolated from DF1 cells using TRIzol reagent (Tiangen). One microgram of total RNA per sample was reverse transcribed into cDNA using FastQuant RT Kit (Tiangen). We next used the ABI Prism 7500 system (Applied Biosystems) in conjunction with One Step SYBR PrimeScript RT-PCR Kit II (TaKaRa) to analyze the expression of UBA52. The expression level of each gene, relative to that for glyceraldehyde-3-phosphate dehydrogenase (GAPDH), was calculated using the 2^-ΔΔct^ method.

### Quantitation of Cytokines and Chemokines

Levels of the selected cytokines and chemokines [interferon (IFN)-β, interleukin (IL)-6, chemokine CC ligand (CCL)4, and tumor necrosis factor (TNF)-α] in the supernatant of infected DF1 cells were determined using ELISA kits (Bio-Swamp). All samples were measured in triplicate, and the experiments were repeated three times independently.

### Statistical Analysis

The viral titers are shown as means ± standard deviations (SD) from three independent experiments. Independent-samples *t*-test was used to analyze the data for TCID50. For all the tests, *p* ≤ 0.05 was considered as being significant.

## Results

### Identification of Proteins From Chicken Cells Interacting With PA-N155 and PA-N182

**FIGURE 1:**
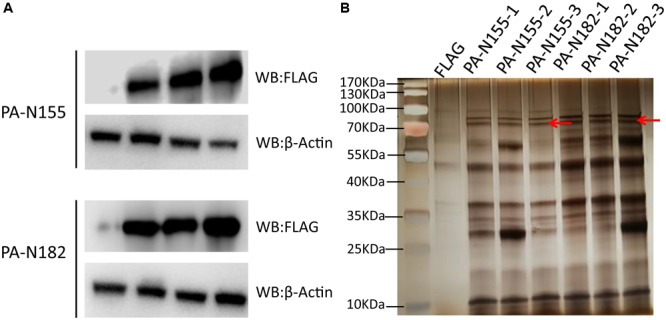
Western blotting and silver staining of exogenously expressed PA-N155 and PA-N182 in chicken cells. **(A)** FLAG-tagged PA-N155 and PA-N182 were transfected into chicken DF1 cells and expression was determined 48 h after transfection. β-Actin was used as a loading control. **(B)** Eluates of triplicate FLAG immunoprecipitates from chicken DF1 cells were subjected to SDS-PAGE and silver stained. FLAG-tagged PA-N155 and PA-N182 proteins are indicated by the red arrows.

The exogenous FLAG-tagged PA-N155 and PA-N182 were highly expressed in chicken DF1 cells, as revealed by western blotting (**Figure 1A**). AP was carried out against the FLAG tag to immunoprecipitate PA-N155 and PA-N182 associated host proteins. Protein complexes pulled down by immunoprecipitation were denatured and separated by SDS-PAGE and the gels were visualized using silver staining (**Figure 1B**). Clear PA-N155 and PA-N182 bands were observed at the expected molecular weight, along with the arrays of immunoprecipitated host proteins. PA-N155, PA-N182 and their interaction partners were not observed in the empty FLAG control, indicating the specific enrichment of PA-N155- and PA-N182-associated proteins.

The interacting proteins that remained after excluding those in the controls and then having a SAINT score > 0.89 were considered to be bona fide PA-N155 or PA-N182 interactors. This resulted in 491 (PA-N155) and 302 (PA-N182) interacting proteins being identified in chicken DF1 cells. Of these, 222 were common host proteins shared by PA-N155 and PA-N182 (Supplementary Tables S1, S2). Several host-interacting proteins are listed in **Table 1**.

**Table 1:** PA-N155- and PA-N182-host interacting proteins in chicken cells (selected proteins).

Viral protein	Host gene symbol	Host protein name
PA-N155	RNF185	E3 ubiquitin-protein ligase RNF185
	PSMC6	26S proteasome regulatory subunit 10B
	LIMA1	LIM domain and actin-binding protein 1
	TRAF2S	TRAF2S protein
	PSMD2	26S proteasome non-ATPase regulatory subunit 2
PA-N182	EIF3D	Eukaryotic translation initiation factor 3 subunit D
	DDX27	Probable ATP-dependent RNA helicase DDX27
	MCM3	DNA replication licensing factor MCM3
	USP9X	Probable ubiquitin carboxyl-terminal hydrolase FAF-X
	XPO1	Exportin-1
PA-N155	COPG	Coatomer subunit gamma-1
PA-N182	IPO5	Importin-5
	UBA52	Ubiquitin-60S ribosomal protein L40
	CCT5	T-complex protein 1 subunit epsilon
	WDR37	WD repeat-containing protein 37

*The SAINT score of the proteins shown in the table > 0.89.*

### PA-N155-and PA-N182-Host Interactomes of H5N1 IAV

**FIGURE 2:**
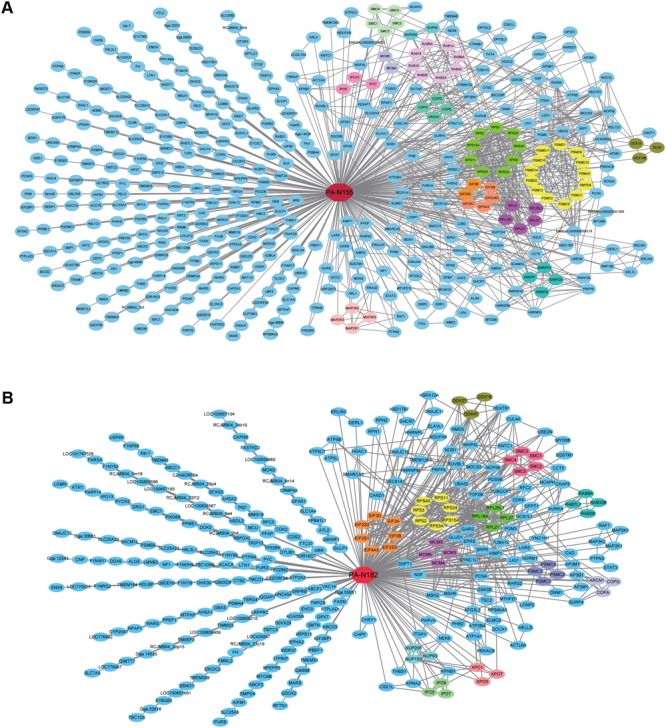
Virus–host predicted interaction network of PA-N155 and PA-N182 in chicken cells. **(A)** PA-N155 host interaction and interactions between PA-N155-associated host proteins are shown. **(B)** PA-N182 host interaction and interactions between PA-N182-associated host proteins are shown. Red nodes in the center of the network represent PA-N155 and PA-N182, and the other nodes represent chicken proteins. Proteins in the same family are often involved in the interactions in the form of complexes, and we expressed them in the same color. Interactions between host factors were incorporated using interaction data from STRING.

To identify the specific protein families that associate with PA-N155 and PA-N182, we analyzed the protein–protein interactions among the PA-N155 and PA-N182 host-interacting factors using the STRING database (Szklarczyk et al., 2017). We then mapped the two predicted interactomes based on MS and STRING analysis by Cytoscape (**Figure 2**).

Many proteins associated with fundamental biological process were identified as PA-N155- and PA-N182-interacting proteins in DF1 chicken cells. Many proteins gathered forming several sub-networks in the PA-N155–host and PA-N182–host interactomes, implying a close relationship among them to form functional protein complexes (**Figure 2**). Most sub-networks were found in both the PA-N155– and PA-N182–host networks, such as Coatomer subunits, and MCM components. MCM3, MCM4, MCM5, and MCM6 were found to interact with PA-N155 and PA-N182 (**Figure 2**). Eukaryotic translation initiation factor (eIF) subunits including eIF2S1, eIF2S3, eIF3B, eIF4A3, eIF2AK2, and eIF3M formed a network interacting with PA-N155 (**Figure 2A**). Similarly, eIF2S1, eIF2S3, eIF3B, eIF4A3, eIF2S2, eIF3A, and eIF3D formed a network interacting with PA-N182 (**Figure 2B**). The ribosomal proteins including RPS2, RPS3, RPS3A, RPS4X, RPS24, RPS15A, RPL18A, RPL26L1, RPL27, and RPL21 also interacted with PA-N155 as a protein complex.

### Gene Ontology and Pathway Enrichment Analysis of PA-N155– and PA-N182–Host Interacting Partners of H5N1 IAV

Gene ontology (GO) analysis of the 491 PA-N155 and 302 PA-N182 host-interacting proteins was done with DAVID. Gene ontology enrichment of PA-N155 and PA-N182 host-interacting proteins included viral process, RNA processing, protein transport and cell cycle. Many other different terms, such as NIK/NF-κB signaling and Ribosome were significantly enriched only in the host proteins interacting with PA-N155 (**Table 2**).

**Table 2:** GO enrichment of PA-N155 and PA-N182 host-interacting proteins.

Virus	GO term	Gene number	*P*-value
PA-N155	*Biological process*		
	Protein transport	94	2.98E-12
	Viral process	50	3.50E-10
	NIK/NF-kappaB signaling	19	1.51E-08
	Translational initiation	20	5.86E-06
	RNA processing	46	2.81E-05
	Cell cycle	61	0.0259
	*Molecular function*		
	RNA binding	83	2.22E-11
	Hydrolase activity	101	4.52E-08
	Nucleotide binding	24	0.00479
	Helicase activity	13	0.0389
	*Cellular component*		
	Proteasome accessory Complex	11	1.87E-09
	Organelle membrane	106	1.53E-07
	Organelle envelope	58	2.15E-07
	Ribosome	18	0.00569
PA-N182	*Biological process*		
	Viral process	40	4.85E-11
	Protein transport	60	3.47E-07
	Cell cycle	53	3.06E-06
	Protein localization	67	1.75E-05
	RNA processing	28	0.0431
	*Molecular function*		
	RNA binding	64	1.80E-12
	Helicase activity	13	0.000214
	Nuclear localization sequence binding	6	0.00189
	Signal sequence binding	7	0.00324
	*Cellular component*		
	Organelle envelope	43	4.34E-07
	Organelle membrane	67	0.000583
	Proteasome accessory complex	5	0.0351

**FIGURE 3:**
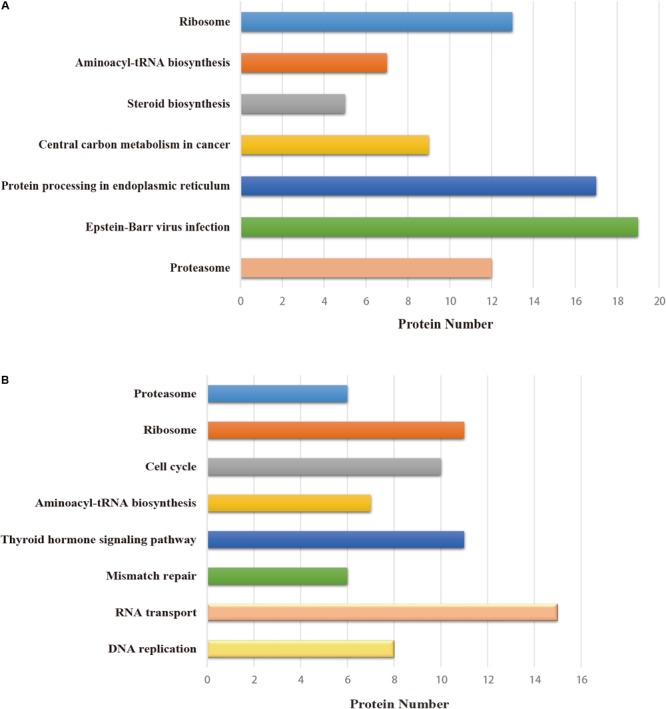
Pathway analysis of the cellular proteins interacting with truncated PA, based on KEGG. **(A)** Pathway enrichment analysis of PA-N155. **(B)** Pathway enrichment analysis of PA-N182. The terms that were significantly enriched (*p* < 0.05) are shown.

All host factors interacting with PA-N155 and PA-N182 were analyzed for pathway enrichment using KOBAS (Xie et al., 2011). Pathways including Epstein–Barr virus infection, protein processing in endoplasmic reticulum, central carbon metabolism in cancer and steroid biosynthesis were significantly enriched in the PA-N155 host-interacting proteins; DNA replication, RNA transport, mismatch repair, thyroid hormone signaling pathway and cell cycle were enriched in the PA-N182 host-interacting proteins (**Figure 3**). Ribosome, aminoacyl-tRNA biosynthesis and proteasome were enriched in the interacting chicken proteins of both viral proteins (**Figure 3**). These results indicated that proteins involved in DNA replication, ribosomes, RNA transport, and proteasomes might be related to IAV infection and participate in IAV replication.

### Domain Analysis of PA-N155 and PA-N182 Host-Interacting Proteins of H5N1 IAV

**FIGURE 4:**
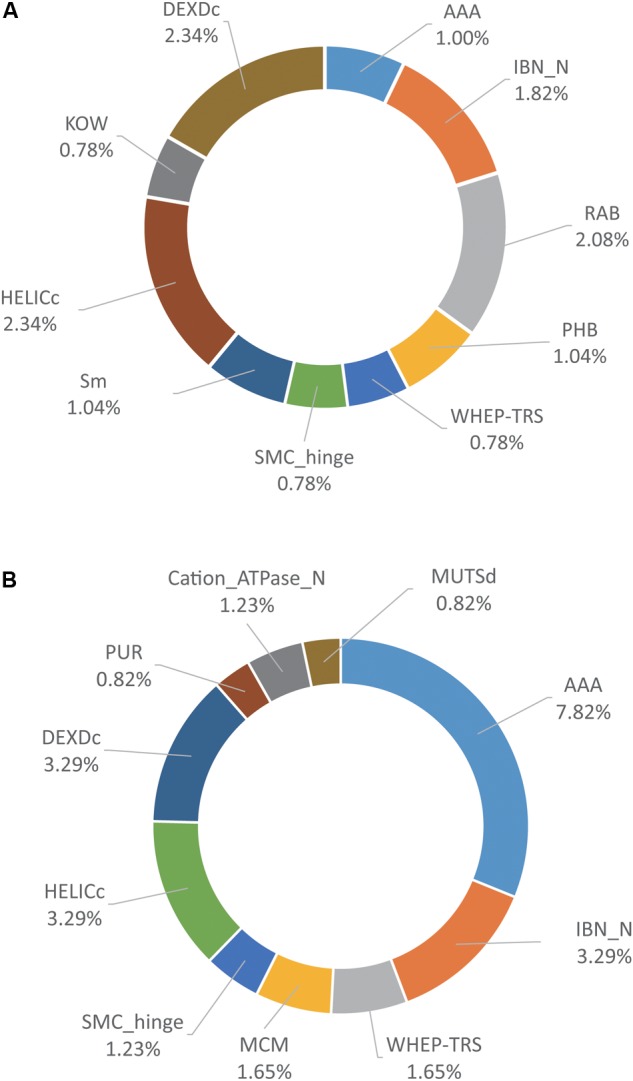
Domain analysis of PA-N155 and PA-N182 host-interacting proteins of H5N1 IAV. **(A)** Domain analysis of PA-N155 host-interacting proteins. **(B)** Domain analysis of PA-N182 host-interacting proteins. The top 10 domains that were significantly enriched are shown.

Protein domain enrichment analysis of the host proteins associated with PA-N155 and PA-N182 using Funrich (Pathan et al., 2015) identified the top 10 enriched protein domains among the PA-N155 and PA-N182 host-interacting proteins (**Figure 4**). A total of 26 protein domains including HELICc, AAA, Cation_ATPase_N, DEXDc, MCM, RAP, and CDC48_2 were over-represented in both sets of host proteins associated with PA-N155 and PA-N182 (Supplementary Table S3). Several protein domains, including RAB, UDPG_MGDP_dh_C and Sm-like domain were enriched only in the host proteins associated with PA-N155 (Supplementary Table S3). Conversely, eIF2B_5, TOP4c, ProRS-C_1 and several other protein domains were enriched among those associated with PA-N182 (Supplementary Table S3). Of particular interest, eIF4A, part of the DEA (D/H)-box RNA helicase family, had the HELICc protein domain that plays an important role in viral replication.

### Comparative Analysis of the Proteins Interacting With PA-N155, PA-N182, and PA

**FIGURE 5:**
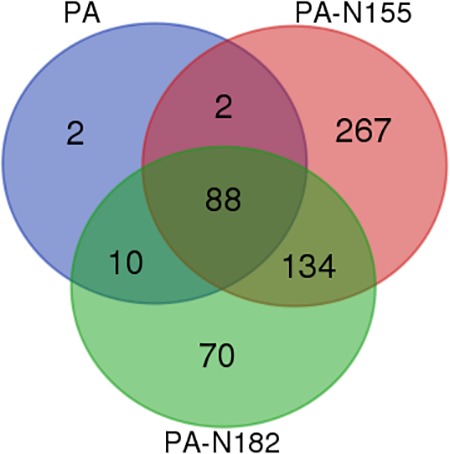
Difference between the interacting host proteins of PA, PA-N155, and PA-N182 of H5N1 IAV. Venn diagram showing shared host-interacting proteins between host factors associated with PA, PA-N155, and PA-N182. The host-interacting factors of PA-N155 and PA-N182 were identified in the present study. The PA-associated proteins were identified by Wang et al. (2016) in chicken cells.

Previous studies have identified 102 PA host-interacting proteins in chicken DF1 cells by AP-MS (Li et al., 2016; Wang et al., 2016). In the present study we compared the virus–host interactomes of these three proteins. There were 88 host-interacting proteins shared by PA, PA-N155, and PA-N182. Microsomal glutathione *S*-transferase 1 and NIMA related kinase 7 were shared by PA and PA-N155. Ten proteins were shared by PA and PA-N182 (**Figure 5** and Supplementary Table S4). E3 ubiquitin-protein ligase (RNF185) only showed a close interaction with PA-N155 (Supplementary Table S4). eIF3D was only found in PA-N182 host-interacting proteins of H5N1 IAV (Supplementary Table S4).

### UBA52 Significantly Influences IAV Replication in Host Cells

**FIGURE 6:**
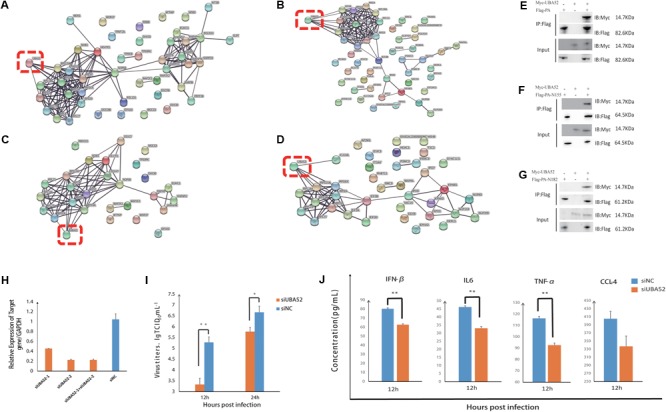
UBA52 influences IAV replication in DF1 cells. **(A)** Interaction network of the identified proteins with PA-N155 that are involved in RNA processing. **(B)** Interaction network of the identified proteins with PA-N155 that are involved in viral processes. **(C)** Interaction network of the identified proteins with PA-N182 involved in RNA processing. **(D)** Interaction network of the identified proteins with PA-N182 involved in viral processes. Red points represent UBA52 and the other colors represent the other identified proteins involved in RNA processing or viral processes. **(E–G)** Co-IP assay confirming the interaction between UBA52 and PA, PA-N155 or PA-N182. **(H)** Knockdown efficiency of UBA52. DF1 cells were transfected with siNC or siUBA52. The result shown is from quantitative PCR performed 24 h after infection. **(I)** Progeny virus titers decreased significantly after transfection of DF1 cells with siUBA52 at 12 and 24 hpi. The results are presented as the mean ± SD from three independent experiments. ^∗^*p* < 0.05 compared with cells transfected with siNC. ^∗∗^*p* < 0.01 compared with cells transfected with siNC. **(J)** Cytokines and chemokines expression in the DF1 cells. Cytokine or chemokine expression was expressed as the mean ± SD concentration. ^∗^*p* < 0.05 compared with cells transfected with siNC. ^∗∗^*p* < 0.01 compared with cells transfected with siNC.

UBA52 (ubiquitinated at the N terminus and ribosomal protein L40 at the C terminus) was shared by PA, PA-N155, and PA-N182. UBA52 occupied an important position in the interaction networks of RNA processing and viral process (**Figures 6A–D** and Supplementary Table S5). UBA52 had a close interaction with the RP family that is vital for virus replication. PA, PA-N155, or PA-N182 was co-transfected with UBA52 into cells and our results demonstrated that UBA52 had interactions with PA, PA-N55, and PA-N182 (**Figures 6E–G**). To determine the effect of UBA52 on the replication of IAV, we knocked down the expression by siRNA (**Figure 6H**). The mixture of the two pairs of siRNA was used in the following experiment. The titers of progeny viruses were quantified after the knock down of UBA52. There was a significant reduction in the progeny viral titer in the supernatant of the UBA52-knockdown cells at 12 and 24 hpi (**Figure 6I**), indicating a promotive role of UBA52 in IAV infection. To further investigate the effect of UBA52-knockdown on immune response upon virus infection, we tested the production of some cytokines and chemokines in the supernatant of infected DF1 cells. The infected UBA52-knockdown cells showed significantly reduced expression of IFN-β, IL-6, TNF-α, and CCL-4 at 12 hpi compared to the control group (**Figure 6J**).

## Discussion

Many studies have characterized the biological functions of the PA proteins, but fewer on the two truncated PA proteins and notably, the virus–host factors remain unknown. PA-N155 and PA-N182 are important for efficient viral replication (Wise et al., 2009). The interactomes of the H5N1 virus PA-N155 and PA-N182 proteins were analyzed here to identify host proteins in chicken cells associated with them. Two sets of controls were used to exclude contaminants as much as possible, including elimination of proteins pulled down from cells transfected with empty FLAG constructions and by normal IgG. Interactions identified as having a high probability of physiological interaction (>0.89) were considered to be authentic partners of PA-N155 and PA-N182. These analyses identified 491 proteins interacting with PA-N155 and 302 with PA-N182 in chicken DF1 cells. In this study, we identified the host proteins that had interaction with PA-N155 and PA-N182 by AP-MS, and we mapped the two predicted interactomes based on STRING analysis by Cytoscape (**Figure 2**). The active interaction sources analyzed by STRING included text mining, experiments, databases, co-expression, neighborhood, gene fusion and co-occurrence, with minimum interaction scores exceeding 0.7. On the other hand, most cellular proteins perform biological functions in the form of functional protein complexes, so whether the interaction between viral proteins and host proteins mentioned above is direct or not remains to be further verified.

WD repeats (MDRs) are minimally conserved regions of ∼40 amino acids typically bracketed by Gly-His and Trp-Asp (GH-WD) that may facilitate the formation of heterotrimeric or multiprotein complexes. Members of this family are involved in a variety of cellular processes, including cell cycle progression, signal transduction, apoptosis, and gene regulation (Claudio et al., 1999; Young et al., 2015). WDR82 negatively regulates viral replication through mediating TNF receptor associated factor 3 (TRAF3) polyubiquitination status and stability in mitochondria (Zhu et al., 2015). WDR5 promotes the activation of the virus-triggered type I IFN signaling pathway involving epigenetic regulation (Wang et al., 2010; Aitken et al., 2016). WDR63 is a microtubule protein that plays an important role in maintaining cell morphology and intracellular material transport (Young et al., 2015). Our study showed that WDR37 interacted with PA-N155 and PA-N182.

RNF185 showed interaction with PA-N155. Much is known of RNF185, especially about its role associated with viral infections. As a mitochondrial outer membrane ubiquitin E3 ligase, RNF185 is involved in the regulation of selective autophagy (Tang et al., 2011). RNF185 is also central to the control of degradation of cystic fibrosis transmembrane conductance regulator (El Khouri et al., 2013). Wang et al. uncovered RNF185 as the first E3 ubiquitin ligase of cGAS, shedding light on the regulation of cGAS activity in the innate immune response (Wang Q. et al., 2017).

Many host factors form a sub-network to interact with viral proteins. MCM3, MCM4, MCM5, and MCM6 were identified to have interactions with both PA-N155 and PA-N182. MCM functions as a scaffold between the nascent RNA chains and viral RNA polymerase (Kawaguchi and Nagata, 2007). MCM2 to MCM7 may regulate the synergistic activation of apoptosis in triple negative breast cancer (Qiu et al., 2017). eIF2 subunits including eIF2S1, eIF2S2 and eIF2S3 and eIF3 subunits including eIF3A, eIF3B, eIF3D, and eIF3M were identified as forming a sub-network that interacted with PA-N155 and PA-N182. Much has been done to find out the role of eIFs, and several proteins have been identified as antiviral host factors. eIF3 is a central player in recruitment of the pre-initiation complex to mRNA, an essential aspect of protein synthesis (Aitken et al., 2016). eIF3D, eIF3E, and eIF3F can inhibit replication of HIV (Jager et al., 2011). eIF1A affects the translation initiation of hepatitis C virus by stabilizing tRNA binding (Jaafar et al., 2016). The present results indicate that eIF3D and other interacting factors may also affect the replication of IAV.

One of the most important finding of our study is that UBA52 interacts with PA, PA-N155, and PA-N182 and promotes the replication of highly pathogenic H5N1 avian IAV. PA contains 716 amino acids, under the action of trypsin, which can be hydrolyzed into two parts: the N-terminal domain of PA (1 to 212 or 213 amino acid residues, PA-N) and the C-terminal domain of PA (212 or 213 to 716 amino acid residues, PA-C) (Fodor et al., 2002). PA-N plays a critical role in protein stability, endonuclease activity, cap binding, and virion RNA promoter binding (Hara et al., 2006). The PA-C is usually associated with N-terminal domain of PB1, which participates in the combination of vRNA or cRNA of IAV (Obayashi et al., 2008). UBA52 consists of ubiquitin at the amino-terminus and RPL40 at the carboxy-terminus (Kobayashi et al., 2016). Ubiquitination is a crucial post-translational modification and RPL40 is essential for cap-dependent translation initiation of vesicular stomatitis virus mRNA (Lee et al., 2012). The whole replication cycle of influenza virus takes place in the nucleus, and the virus needs the host RNA splicing machinery to splice viral mRNA (Herz et al., 1981). UBA52 interacts with the RP family, which serves as a significant component in viral processing. There was a significant reduction in the progeny viral titer in the conditioned culture media of the UBA52-knockdown cells at 12 and 24 hpi. UBA52-knockdown reduced the viral titer and further significantly reduced levels of IFN-β, IL-6, TNF-α, and CCL-4 when compared to the parental group. Here we demonstrate that UBA52 interacts with PA, PA-N155 and PA-N182, indicating UBA52 may interact with the PA-C for promoting the replication of highly pathogenic H5N1 avian IAV.

## Author Contributions

QW, QL, TL, JW, DP, and GZ: conceived and designed the experiments. QW, QL, and ZS: performed the experiments. QW and QL: analyzed the data. ZG, FW, XY, RL, MZ, HC, GbC, GhC, HZ, and HL: contributed reagents/materials/analysis tools. QW, QL, and GZ: wrote the paper.

## Conflict of Interest Statement

The authors declare that the research was conducted in the absence of any commercial or financial relationships that could be construed as a potential conflict of interest.
